# How to Link Assessment and Suitable Interventions for Adolescents: Relationships among Mental Health, Friendships, Demographic Indicators and Well-Being at School

**DOI:** 10.3390/children11080939

**Published:** 2024-08-03

**Authors:** Rokas Šambaras, Agnė Butvilaitė, Justina Andruškevič, Natalja Istomina, Sigita Lesinskienė

**Affiliations:** 1 Clinic of Psychiatry, Institute of Clinical Medicine, Faculty of Medicine, Vilnius University, 01513 Vilnius, Lithuania; sigita.lesinskiene@mf.vu.lt; 2 Faculty of Medicine, Vilnius University, 01513 Vilnius, Lithuania; agne.butvilaite@mf.stud.vu.lt (A.B.); justina.andruskevic@mf.stud.vu.lt (J.A.); 3 Institute of Health Sciences, Faculty of Medicine, Vilnius University, 01513 Vilnius, Lithuania; natalja.istomina@mf.vu.lt

**Keywords:** adolescents, mental health, well-being, school, friendships, interventions, internalizing difficulties, externalizing difficulties

## Abstract

Background: Over the last 10 years, a noticeable deterioration in mental health has affected adolescents’ lives. Methods: This study was conducted in 12 secondary schools and 14 high schools located in different cities across Lithuania from October to December 2023. The survey included students aged 12 to 17 years. The adolescents filled out a questionnaire about the need for outside help, friendships, and well-being at school and the Strengths and Difficulties Questionnaire. The goal of our study was to analyze the risk factors that influence adolescents’ psycho-emotional state. Results: A total of 4124 students were involved in this study, with a mean age of 14.48 ± 1.15 years. The sample consisted of 50.9% males and 49.1% females. The boys showed a statistically significantly lower total difficulty score on the Strength and Difficulties Questionnaire (SDQ TDS) and internalizing score (IS) than the girls (12.45 ± 5.39 vs. 14.93 ± 5.76; 5.39 ± 3.30 vs. 7.49 ± 3.64, *p* < 0.001). Also, the SDQ TDS, IS, and externalizing score (ES) were statistically significantly lower in the group of adolescents who lived with both parents (13.50 ± 5.69 vs. 14.76 ± 5.72; 6.32 ± 3.61 vs. 6.98 ± 3.65; 7.18 ± 3.23 vs. 7.78 ± 3.35, *p* < 0.001). Linear regression analysis of the SDQ TDS (R^2^ = 0.406) indicated a significant impact on the feeling of safety at schools (β = −0.173, *p* < 0.001) and loneliness (β = 0.314, *p* < 0.001). Linear regression of the SDQ IS (R^2^ = 0.469) showed that there was a significant predictor effect of bullying (β = 0.170, *p* < 0.001) and loneliness (β = 0.345, *p* < 0.001). However, the linear regression of the SDQ ES (R^2^ = 0.256) showed that there was a significant predictor effect of the feeling of teacher care (β = −0.163, *p* < 0.001) and loneliness (β = 0.166, *p* < 0.001). We determined that gender (OR = 2.30) and loneliness (OR = 1.77) were the most significant factors associated with adolescents seeking help. Conclusions: It is crucial to determine specific risk factors and particular groups of teenagers who need psycho-emotional support the most. The findings of this study may offer valuable insights for advancing additional prevention or support programs aimed at adolescents within higher-risk groups.

## 1. Introduction

Currently, adolescent mental health is a crucial issue. In the last 15 years, there has been a noticeable deterioration in mental health, affecting adolescents’ lives [1]. Based on the 2019–2020 National Mental Health Development Report in China, among 15,280 students in grades 4 to 12, 17.2% had experienced mild depression and 7.4% had experienced severe depression [2]. According to another cross-sectional study, more than one out of ten children in Sweden have low emotional health and face mental health issues [3]. In recent years, experts have become increasingly concerned about adolescent mental health. This phenomenon is attributed to the widespread occurrence of mental health problems within this age cohort and to the fact that a significant number of these disorders manifest prior to the age of 14 [4,5]. Early signs of mental health issues are associated with more significant psychological health problems in the future. For instance, higher levels of irritability at a younger age could lead to an increased risk of avoidance, emotional dysregulation, negative coping strategies, and a higher probability of depression and self-harm in adolescence [6]. Observing the extent of this problem, it is crucial to notice and encourage teenagers to seek help promptly and to make the means of help appealing so that children can choose to seek it.

It is clear that teenagers are at an increased risk of developing psycho-emotional issues, especially when there is a lack of appropriate care. This problem became significantly relevant following the COVID-19 pandemic [7]. Many children experienced various mental health issues during the lockdown period and lacked access to high-quality healthcare. According to a survey conducted in the United States in 2020, one in ten families faced challenges with children experiencing worsened mental health during the pandemic. The article revealed an escalation in anxiety and depressive disorders among youth [8]. Some surveys suggest that up to 37% of adolescents experienced psycho-emotional health challenges that were exacerbated by the pandemic due to social distancing and isolation [9]. Financial issues, complicated relationships with family members, altered circadian rhythms, a paucity of physical activity, and an unhealthy diet also contribute to a worsened psycho-emotional state.

It is essential to thoroughly examine the various strategies, methods, and programs published to assess children’s and youth’s well-being, especially considering the adverse effects of COVID-19 and aggravated psycho-emotional well-being. For example, a project known as SESSAMO aims to evaluate the relationships among lifestyle factors, social influences, and the overall emotional and somatic well-being of teenagers living in Spain between the ages of 14 and 16. The level of posttraumatic stress disorder due to the pandemic was evaluated through the Brief COVID-19 Screen for Child/Adolescent (BCSCA) PTSD, non-suicidal self-injury was analyzed with the Self-Injurious Thoughts and Behaviors Interview (SITBI), and suicide risk was evaluated with the Columbia Suicide Severity Rating Scale (C-SSRS) (screening version). Additionally, the Depression, Anxiety, and Stress Scale (DASS-21), the Children’s Eating Attitudes Test (ChEAT), the Scale of Problematic Internet Use in Adolescents (EUPI-a), and the Game Addiction Scale for Adolescents (GASA) were used. It was proven that the adverse impact of COVID-19 on children’s mental well-being is still immense [9]. It is crucial to not only examine the occurrence of psyche-emotional health challenges among adolescents but also to motivate them to participate in creating different tools for enhancing mental health. In today’s era of artificial intelligence, technology has become an essential component of healthcare. Moreover, it is beneficial when there is a lack of qualified medical help, and it is attractive to teenagers. C. Kostenius and a team of researchers explored the experiences of using the ChatPal chatbot to improve mental well-being. The study revealed that adolescents enjoyed its accessibility, reliability, and the possibility of being anonymous. Furthermore, the surveyed teenagers recommended that reduced loneliness and increased satisfaction could be achieved with the chatbot app by incorporating social and multimodal interactions [10].

Another critical point is that every stage of development is associated with typical manifestations of mental problems for that age. For example, phobias and separation anxiety are known to usually start in childhood, whereas social anxiety disorder emerges in older children and adolescents. Additionally, panic disorder, agoraphobia, depression, and generalized anxiety mostly have their onset in the teen years and young adulthood [5,11]. The contributing factors linked to psycho-emotional health issues are widely recognized and encompass various forms of adversity, such as childhood sexual and physical abuse, familial, societal, and community-based violence, economic hardship, social marginalization, and educational inequities [12]. While the focus on adolescent mental health is welcomed, there is an urgent need to conduct more research aimed at gaining a deeper understanding of the root causes behind recent trends in occurrence and manifestation. In addition, family structure is also a consequential factor in adolescents’ mental health. The rising incidence of partnership dissolution, such as divorces and separations, frequently results in a departure from the traditional family, in which children reside with both birth parents. This shift has led to a proliferation of non-traditional or alternative family configurations, including single-parent households, in which a child resides with only one biological parent; stepfamilies, where partners have children from prior relationships, as well as another prevalent arrangements, such as grandparent-headed families, co-parenting arrangements, and adoptive and foster families. The increasing prevalence of these diverse family structures necessitates a thorough examination of their influence on the children’s well-being [13]. This understanding is essential for developing effective prevention strategies [14]. Considering all of the current issues with adolescents’ psycho-emotional well-being, our study aimed to explore the risk indicators affecting adolescents’ mental health and to enhance our understanding and knowledge regarding these risk factors.

Considering all of the current issues with adolescents’ psycho-emotional well-being, our study aims to explore the factors contributing to adolescents’ mental health risks and find what kind of help might be most acceptable for students experiencing psycho-emotional difficulties. We hypothesize that adolescent girls and boys might differ in seeking help and experiencing psycho-emotional difficulties. In addition, we considered that adolescent help-seeking might be associated with psycho-emotional difficulties, friendships, demographic indicators (city size, family structure), and well-being at school. We assumed that friendships, demographic indicators (size of the city, family structure), and well-being at school would impact adolescents’ psycho-emotional difficulties.

## 2. Materials and Methods

### 2.1. Study Design

The study took place in Lithuania, a nation recognized as developed with a high-income and advanced economy. It is currently placed 37th on the Human Development Index [15]. Lithuania is the most populous country in the Baltic region, with a total population of 2,867,725. In 2023, according to the Statistics Department of Lithuania (SDL), there were 503,510 children between the ages of 0 and 17 residing in Lithuania. In conducting this study, we aimed to cover as wide a range of areas as possible in Lithuania. For this study, schools were selected at random from various geographical locations across Lithuania, including the eastern, northern, western, and southern regions. In our random selection of schools, we included the consideration of whether the schools were located in urban or rural areas. To determine the minimum sample size for our study population of Lithuanian adolescents aged 12 to 17 (*n* = 237,861), we conducted a sample size analysis using a 95% confidence level, 0.5 standard deviation, and a margin of error (confidence interval) of ±5%. Based on the analysis, a minimum of 608 respondents was needed for a satisfactory sample size.

As part of our research, we carried out surveys of students in the main cities and rural regions. To provide clarity, the cities were classified as follows: (1) large cities (population > 100,000); (2) cities that are district centers (population of 5000 to 100,000); and (3) rural areas (population < 5000). 1. Vilnius is the capital and largest city of Lithuania. Based on SDL data, the population of Vilnius in 2024 was 633,917, including 76,686 children aged 7 to 17. Klaipėda, which is situated in western Lithuania, is the largest city in the region. As reported by the SDL in 2024, the city’s population totaled 172,031 people, including 20,870 children between the ages of 7 and 17. 2. Gargždai is a city in the Klaipėda district. Based on the SDL data, it had a population of 15,072, and there were 1984 children (7–17 years old). Kelmė is a city in northwestern Lithuania. According to the SDL, in 2024, it had a population of 9813 and 1226 children aged 7 to 17. Kaišiadorys is a city in central Lithuania. According to the SDL, it had a population of 8334, with 1041 children (7–17 years old). Moletai is one of the larger cities in the eastern part of the country. Based on the SDL data, it had a population of 5783, with 811 children (7–17 years old). Skuodas is a city in the northwestern part of the country. According to the SDL, it had a population of 5391, with 712 children (7–17 years old). 3. Rietavas is a city in western Lithuania. According to the SDL, it had a population of 3234, with 350 children (7–17 years old). Tverai is a small town in the Rietavas district. Juodšiliai is a small town in the Vilnius district. Kražiai, Tytuvėnai, Kražantė, Elvyrava, Vaiguva, Lioliai, and Šaukėnai are located in the Kelmė district. Giedraičiai, Alanta, and Suginčiai are located in the Molėtai district. Rumšiškės, Žiežmariai, Žasliai, and Kruonis are located in the Kaišiadori district. Veiveržėnai is located in the Klaipėda district. To conduct the research, it was determined that 2 secondary and 2 high school students in each large city would participate. In cities that are the centers of districts and rural areas, 1 or 2 secondary and 1 or 2 high school students would participate, depending on whether there were 2 schools in that city.

### 2.2. Participants and Procedure

In the study, a total of 25 educational institutions participated, comprising 12 secondary schools and 14 high schools. An anonymous survey was administered through questionnaires to collect data from students in grades 7 to 10. The specified range corresponded to individuals aged 12 to 17, explicitly targeting adolescents within this age group.

In the first stage, the researchers applied to the Ethics Committee of the Vilnius University Faculty of Medicine and the Institute of Public Health for ethical approval to conduct the study. Later, we sought permission from the Ministry of Education, Science, and Sport of Lithuania to conduct the study in Lithuanian schools. Lithuanian schools were selected based on their urban population and geographic placement. In September 2023, all selected school administrations were invited to participate in an anonymous survey and agreed to take part in the study. Following the ongoing investigation, the school administration promptly notified the students’ parents and obtained their formal consent. After obtaining parental consent, the school staff scheduled a date to administer the questionnaires to the students participating in the study. Researchers visited selected schools from October to December 2023 to interview 7th- to 10th-grade students. Adolescents who had obtained parental consent were provided with the questionnaires and received comprehensive information regarding the study’s objectives, the voluntary nature of participation, and the option to withdraw from the study at any point. The response rate for our study was 79.2%, exhibiting a range from 62.1% to 92.3% across different schools. The students completed the questionnaires in the classroom environment, a task that required approximately 45 min. This statement clearly outlined the study’s goals and underscored our commitment to upholding ethical standards. It highlighted the voluntary nature of participation and the assurance of anonymity and confidentiality. Some adolescents did not provide answers to specific questions, so we did not include unfilled questionnaires or possibly false answers in the study.

### 2.3. Questionnaire

The authors created an anonymous survey questionnaire comprising five parts. The sections were thoughtfully prepared and labeled to ensure that respondents could easily navigate and understand the content.

The questions were separated into five parts:

(1) Socio-demographic information, which consisted of questions about age, gender (female or male), the city in which the school was located, the grade in which the student was studying (7, 8, 9, or 10), and family composition (living with both parents or living with one parent).
(2) The need for outside help: The students were questioned regarding the necessity of outside help, as follows. Within the last six months, have you ever felt the need for outside help with your problems, feelings, behavior, or emotional trouble? (No, I have not felt the need, I have considered getting outside help, or I have sought outside help). If you have sought help, where/whose help? (Students were able to enter a free-form area where they could seek outside help).
(3) Friendships: Students were asked the following. How many close friends do you have? (no friends, only one friend, 2 friends, >3 friends). Over the last 12 months, how frequently have you experienced feelings of loneliness? (Never, rarely, sometimes, most of the time, or always).
(4) Well-being at school: Students were presented with the following. How frequently in the last six months have you experienced bullying at school? (not at all, <1 per week, >1 per week, or most days). I feel safe at school (never, sometimes, often, or always). Teachers care about me (never, sometimes, often, always).
(5) The Strengths and Difficulties Questionnaire (SDQ) for children and adolescents was used to assess psycho-emotional difficulties [16]. The questionnaire used in our research has been validated in Lithuania and is widely employed in diagnostic research and clinical practice [17]. The tool offers an efficient and user-friendly approach for appraising the mental health issues of adolescents. The self-reported version of the Strengths and Difficulties Questionnaire (SDQ) includes 25 statements that assess positive and negative characteristics in adolescents. The statements provided constitute five scales, each consisting of five statements. These scales address hyperactivity and inattention, emotional difficulties, conduct, and peer-related difficulties, as well as prosocial behavior. Ten statements have been formulated to delineate adolescent strengths, whereas fifteen have been formulated to capture adolescent difficulties. Adolescents assess each statement by reflecting on three potential responses: not true, somewhat true, and certainly true. The total difficulty score (TDS) is calculated by summing the scores of all scales, excluding prosocial behavior. The range of scale estimates can be from 0 to 40 points. Higher estimates are indicative of increased emotional and behavioral difficulties. In this study’s procedure, we used the internalizing score (IS) and externalizing score (ES). The scale of internalized difficulties is determined by combining emotional and peer-related difficulties, while the subscale of externalized difficulties comprises conduct and hyperactivity scales. The range for both internalized and externalized scale estimates is from 0 to 20. A higher sum of the scale scores indicates a greater degree of psycho-emotional difficulties in adolescents.

The overall internal consistency coefficient for the total difficulty score was acceptable at 0.72. Cronbach’s α values for emotional symptoms were 0.73, peer problems 0.71, hyperactivity 0.70, and the conduct problem scale 0.69. However, the prosocial behavior scale demonstrated the lowest Cronbach α value at 0.61.

### 2.4. Statistical Analysis

The data were manually coded using Microsoft Excel to assign numerical values to the corresponding responses. Subsequently, statistical data analysis was conducted using Microsoft Excel 2010 and IBM SPSS 26.0 programs. A comprehensive statistical analysis was carried out, taking into account key background variables such as the city in which the school was located and the need for outside help. The additional variables were student psycho-emotional difficulties, friendships, and well-being at school. The background and additional data were analyzed with quantitative data, namely, the SDQ total difficulty score and the internalizing and externalizing scoring of SDQ. Quantitative variables conforming to a normal distribution were characterized using the mean and standard deviation (SD). Assessment of the normality of the variables’ distribution was conducted through the Shapiro–Wilk test. The study examined the disparities among background variables by employing one-way analysis of variance (ANOVA) with more than two separate groups. A comparison of quantitative variables between groups was performed utilizing the post hoc Tukey test. Spearman’s rank correlation test was employed to ascertain the associations between quantitative variables and ranked variables. Linear regression analysis was conducted to account for the variables while evaluating the relationships between the primary background variables, additional variables, and the SDQ total difficulty score, internalizing score, and externalizing score. The essential criteria for a suitable linear regression model enabling the deduction of valid conclusions encompassed a determination coefficient (R^2^) exceeding 0.20 and an ANOVA *p*-value below 0.05. The variance inflation factor (VIF) was used to check for multicollinearity, with values required to be ≤4. A binary regression model was used to achieve a good fit for the data (Nagelkerke’s R^2^ > 0.20 and VIF ≤ 5). The results are presented as odds ratios (ORs) with a 95% confidence interval (CI). Statistical significance was established at a significance level of *p* < 0.05.

## 3. Results

### 3.1. Main Sample Characteristics

This research involved 4124 students. The adolescents’ mean age was 14.48 ± 1.15 years, with a minimum age of 12 years and a maximum age of 17 years. Both genders were evenly distributed within the sample, which comprised 2037 (50.9%) males and 1965 (49.1%) females. A total of 1644 (39.9%) of the interviewed students were from large cities (population > 100,000). The distribution of students across classes showed notable consistency, with percentages ranging from 23.9% to 26.4%. A total of 1186 adolescents—about 83.3% of them—lived with both parents. Table 1 presents the sample’s characteristics.

### 3.2. Help-Seeking among Students

**Figure 1:**
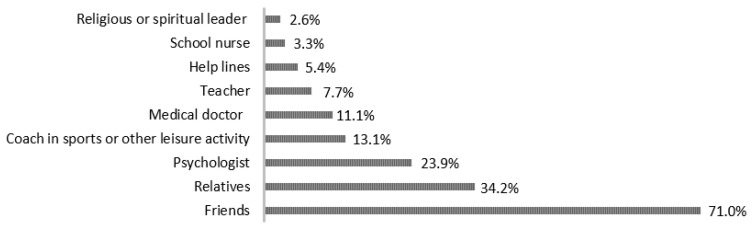
Where students most often sought help for their problems, feelings, behavior, or emotional troubles.

In the previous six months, one out of ten students (389; 9.4%) sought outside help. In addition, one-third of the students (1279; 31.0%) indicated that they had considered seeking outside help. A total of 2456 (59.6%) students stated that they did not feel the need for outside help. Students who sought outside help for their problems, feelings, behavior, or emotional trouble in the previous six months indicated that they usually sought help from their friends (276 (71.0%)). Figure 1 indicates where students most often sought help for their problems, feelings, behavior, or emotional troubles.

### 3.3. Students’ Well-Being at School and Friendships

In the previous 12 months, more than half of the students never (1080; 26.6%) or rarely (1145; 28.2%) felt lonely. Two-thirds of the students (2675; 65.9%) indicated that they had more than three friends. A total of 3003 (74.2%) students stated that they were not bullied at school in the last 6 months, and 1193 (28.9%) always felt safe at school. However, only 499 (12.1%) students indicated that teachers cared about them. The data detailing the attributes of students’ well-being at school and their friendships are shown in Table 2.

### 3.4. Students’ Psycho-Emotional Problems

In our study, we noticed that the mean SDQ total difficulty score of all adolescents was 13.73 ± 5.73. In addition, the analysis of the internalizing score (IS) and externalizing score (ES) showed that the mean IS was 6.44 ± 3.62 and the mean ES was 7.29 ± 3.26. After performing an independent-sample *t*-test and comparing the SDQ TDSs between genders, it was found that the average SDQ TDS for female participants (14.89 ± 5.75) was significantly higher than that for male participants (12.45 ± 5.35), (*p* < 0.001). Identical findings were achieved when comparing the SDQ IS (7.47 ± 3.64 vs. 5.38 ± 3.29) with a *p*-value of less than 0.001. No statistically significant difference was observed in the comparison of the SDQ scores with the size of the city in which the students were studying. However, the largest SDQ TDS (13.91), SDQ IS (6.53), and SDQ ES (7.38) estimates were found in large cities (population > 100,000). In addition, the SDQ TDS, IS, and ES were statistically significantly lower in the group of adolescents who lived with both parents (*p* < 0.001). Table 3 presents a more extensive comparison of adolescents’ psycho-emotional problems based on gender, city of residence, and family composition.

### 3.5. Students’ Psycho-Emotional Problems and Help-Seeking

A one-way analysis of variance (ANOVA) was used to compare the SDQ TDS and students’ help-seeking behavior. The study revealed a statistically significant disparity among adolescents who sought help, considered seeking it, or did not seek help at all. Post hoc pairwise comparisons revealed that individuals who sought external help for their psycho-emotional difficulties exhibited significantly higher SDQ TDSs than those who did not require external help (*p* < 0.001). Furthermore, a moderate positive correlation was found between adolescents’ SDQ total difficulty scores and help-seeking (ρ_s_ = 0.405, *p* < 0.001). The comparison of the SDQ IS and help-seeking delivered similar results. Comparisons showed that those who sought external help for their psycho-emotional difficulties had higher SDQ ISs than those who indicated that they did not need external help (*p* < 0.001). In addition, a moderate positive correlation was found between adolescents’ SDQ internalizing score and help-seeking (ρ_s_ = 0.430, *p* < 0.001). Nevertheless, the difference between the SDQ externalizing score of students who considered seeking outside help and those who sought external help was not statistically significant (*p* = 0.091). On the other hand, we observed a weak positive correlation between the SDQ externalizing score and help-seeking (ρ_s_ = 0.405, *p* < 0.001). In Table 4, a thorough comparison of students’ psycho-emotional challenges and their engagement in help-seeking behavior is presented.

### 3.6. Students’ Emotional Problems, Behavioral Problems, and Well-Being at School

A one-way analysis of variance (ANOVA) was used to examine the association between the SDQ total difficulty score and adolescents faced with bullying over the previous six months. The analysis revealed a statistically significant disparity in the incidence of bullying. After conducting post hoc pairwise comparisons, it was evident that individuals subjected to bullying exhibited notably higher SDQ TDSs than those who reported no history of bullying (*p* < 0.001). The analysis revealed a weak positive correlation between adolescents’ SDQ TDSs and the frequency of bullying (ρ_s_ = 0.328, *p* < 0.001). The SDQ internalizing score and bullying frequency comparison had quite similar results. Those who were bullied had higher SDQ ISs than those who did not experience bullying at all (*p* < 0.001). In addition, a moderate positive correlation was observed between adolescents’ SDQ internalizing scores and bullying frequency (ρ_s_ = 0.341, *p* < 0.001). The study found no statistically significant variance in the SDQ externalizing scores among adolescents who experienced bullying less than once per week, more than once per week, or most days (*p* = 0.112, *p* = 0.062). On the other hand, we observed a weak positive correlation between adolescents’ SDQ ESs and bullying frequency (ρ_s_ = 0.197, *p* < 0.001).

Analogous results were obtained when comparing the SDQ total difficulty score and the SDQ internalizing and externalizing scores with the student’s sense of safety at school (*p* < 0.001). Following post hoc pairwise comparisons, it was determined that adolescents who reported feeling unsafe at school exhibited notably higher SDQ total difficulty, SDQ internalizing, and SDQ externalizing scores compared to students who noted feeling safe at school (*p* < 0.001). Furthermore, weak negative correlations were observed between the SDQ TDS, IS, and ES and the perceived feeling of safety at school (ρ_s_ = −0.397, *p* < 0.001; ρ_s_ = −0.372, *p* < 0.001; ρ_s_ = −0.285, *p* < 0.001).

In addition, it was observed that students who reported feeling that their teachers cared about them had lower SDQ TDSs and SDQ ISs than adolescents who reported feeling that their teachers did not care about them (*p* < 0.001; *p* < 0.001). There was no statistically significant difference in the Strengths and Difficulties Questionnaire (SDQ) emotional symptoms between students who expressed that they felt teachers cared for them often and always (*p* = 0.174). Likewise, weak statistically significant negative correlations were found between the SDQ TDS, IS, and ES and the feeling of teacher care (ρ_s_ = −0.321, *p* < 0.001; ρ_s_ = −0.247, *p* < 0.001; ρ_s_ = −0.283, *p* < 0.001). Complete information on students’ psycho-emotional problems and their association with their well-being at school is given in Table 5.

### 3.7. Students’ Psycho-Emotional Problems and Friendship

Adolescents who reported feeling lonely in the last 12 months had statistically significantly higher SDQ TDSs, SDQ ISs, and SDQ ESs than students who had never felt lonely during the previous 12 months (*p* < 0.001). A moderate statistically significant positive correlation was observed between the frequency of experiencing loneliness in the last 12 months and the SDQ TDSs and SDQ ISs (ρ_s_ = 0.520, *p* < 0.001; ρ_s_ = 0.568, *p* < 0.001). In addition, a weak statistically significant positive correlation was observed between the frequency of experiencing loneliness and the SDQ ES (ρ_s_ = 0.285, *p* < 0.001).

Similar results were obtained when the SDQ TDSs and SDQ ISs were compared with the number of real friends that adolescents had (*p* < 0.001). Post hoc pairwise comparisons showed that the SDQ TDSs and SDQ ISs of students with a higher number of true friends were lower than those of students with no friends (*p* < 0.001). In addition, a weak statistically significant negative correlation was observed between the numbers of friends and the SDQ TDSs and SDQ ISs (ρ_s_ = −0.224, *p* < 0.001; ρ_s_ = −0.310, *p* < 0.001). However, there was no significant difference between students’ SDQ ESs according to their number of friends (*p* = 0.053). In addition, we observed a very weak statistically significant negative correlation (ρ_s_ = −0.047, *p* < 0.001). Table 6 compares students’ emotional and behavioral problems and friendships more comprehensively.

### 3.8. Factors Associated with Students’ Emotional and Behavioral Problems

The linear regression model met the required parameters when the dependent variables were the SDQ TDS, SDQ IS, and SDQ ES and the regressors were student gender, cities, help-seeking, bullying, a feeling of safety at schools, a feeling of teacher care, loneliness, and friends. A further linear regression of the SDQ TDS (determination coefficient R^2^ = 0.406, VIF for all factors was ≤5) showed a significant effect for the predictor of a feeling of safety at schools (β standardized coefficient = −0.173, *p* < 0.001) and a more significant effect for the predictor of loneliness (β standardized coefficient = 0.314, *p* < 0.001). Linear regression of the SDQ IS (coefficient of determination R^2^ = 0.469, VIF ≤ 5) showed a significant predictor effect of bullying (β standardized coefficient = 0.170, *p* < 0.001) and a more significant effect for the predictor of loneliness (β standardized coefficient = 0.345, *p* < 0.001). However, linear regression of the SDQ ES (co-efficient of determination R^2^ = 0.256, VIF ≤ 5) showed a significant predictor effect for a feeling of teacher care (β standardized coefficient = −0.163, *p* < 0.001) and a more significant effect for the predictor of loneliness (β standardized coefficient = 0.166, *p* < 0.001). Still, the impact of loneliness was more significant for the SDQ IS than for the SDQ ES. Similar results showed that a feeling of teacher care was a more significant regressor for the SDQ ES than for the SDQ IS. Table 7 presents the complete regression analysis of the SDQ TDS, SDQ IS, and SDQ ES.

### 3.9. Factors Associated with Students’ Help-Seeking

Binary logistic regression was performed to select the most significant variables. Nagelkerke’s R^2^ coefficient of determination was 0.353 and VIF for all factors was ≤5, so we considered the data suitable for constructing a regression model. For the data to be suitable for binary logistic regression, we distinguished two groups: one group of students who did not need outside help and another group of students who considered seeking outside help or had sought outside help. We determined that gender (OR = 2.30), loneliness (OR = 1.77), and SDQ IS (OR = 1.10) were the most significant factors associated with adolescent help-seeking. More detailed associations between adolescent help-seeking and the variables are given in Table 8.

## 4. Discussion

Our research showed that in the previous six months, 10% of students had sought external support for issues related to their problems, emotions, behavior, or emotional distress. One-third of the students indicated that they had considered seeking outside help. Most of the time, teenagers sought help from their friends. In our study, students’ help-seeking was associated with gender and experiences of loneliness. Seeking support for mental health issues during adolescence, when most related disorders first appear, can lead to better outcomes than seeking intervention later in life [18]. Early intervention plays a pivotal role in attaining favorable mental health outcomes, such as lowering suicide rates [18]. Research consistently indicates that adolescents strongly prefer receiving support from friends and parents than from psychologists and doctors, irrespective of their gender, age, or where they reside [19]. During early adolescence, both boys and girls exhibit similar tendencies in seeking assistance. However, research indicates that as boys progress through adolescence, they demonstrate an increasing reluctance to seek help in comparison to girls [20]. This indicates that adolescent boys’ reluctance to express their feelings and emotions to others may hinder their help-seeking behavior. This finding underscores the real-world effects of gender role socialization, indicating that adolescent girls might be more inclined to openly address their symptoms and articulate their emotions and requirements [21].

According to our research findings, 12- to 17-year-old girls exhibited notably higher internalizing difficulty scores and total SDQ scores than boys of the same age. In a 2021 analysis, notable gender disparities were identified concerning externalizing and internalizing problems. Specifically, boys more often exhibited acting-out behaviors in comparison to girls, while girls demonstrated more internalizing behaviors [22]. In research conducted in the Netherlands, gender comparisons were undertaken, particularly among the age groups 6–11 and 12–18. The results revealed that male subjects generally exhibited lower SDQ scores compared to their female counterparts. The investigation unearthed conspicuous gender disparities in hyperactivity–inattention, peer-related issues, prosocial conduct, externalizing behaviors, and overall adversity [23]. Conversely, comparing genders, male adolescents reported significantly higher levels of loneliness, hyperactivity, conduct issues, and social relationship problems than females. Female adolescents, on the other hand, disclosed considerably more emotional symptoms and demonstrated more positive social behaviors than the opposite gender did [24]. However, a study in the United States found no substantial differences across the scales of conduct problems, hyperactivity, peer problems, or emotional symptoms between males and females [25].

Our study revealed that adolescents living with one parent experienced greater emotional and behavioral difficulties than those living with both parents. Children’s ability to learn, behavioral patterns, and mental health depend on early experiences, resources, and family socioeconomic status. The latter is known to be one of the key determinants impacting children’s successful development. A poorer level of parents’ education, marital crisis, unfavorable living conditions, unemployment, and low income are linked to an increased likelihood of emotional and behavioral issues and psychological well-being concerns in offspring. Moreover, this association is more robust and has a more significant impact on children than on adults. This means that children coming from households with low socioeconomic status tend to have more mental health problems [2]. Persistent poverty was linked to the highest increase in the likelihood of behavioral problems at age 11, in contrast to never experiencing poverty. This association was strong for internalizing behaviors, but less pronounced for externalizing problems [26]. Children from stepparent families had significantly greater total difficulty scores, along with elevated scores on the Conduct Problems and Emotional Symptoms scales, in comparison with youngsters from nuclear families. Additionally, those who came from single-parent households demonstrated elevated total points across all problem scales of the SDQ [13]. Children from blended families had substantially greater total difficulty scores, along with higher scores on the Conduct Problems and Emotional Symptoms scales, in comparison with children from nuclear families. Additionally, participants from single-parent households also demonstrated higher scores across all problem scales of the SDQ [13].

Our study showed that one of the strongest factors associated with poorer psycho-emotional health among students was the feeling of loneliness. Loneliness is recognized as a contributing factor to worsened mental health, cognitive dysfunction, sleeping disorders, and more significant physical health problems. Adolescents must be a part of a community and build close relationships with peers because they start to explore their own independence and often prioritize friendship over family [5]. Many sources indicate that the problem of loneliness has been rapidly increasing within the younger population [27]. The coronavirus pandemic and the increased prevalence of addiction to social media even aggravated the situation. The lack of social interaction and sensitivity to social rejection can result in a range of physical and mental health challenges. Prior research indicates that friendships are vital for the development of adolescents. As adolescents grow, friends become more significant, and they often prioritize these relationships over others as they spend more time with them [28]. According to Manfro’s study, higher scores in the emotion, hyperkinetic, and conduct domains were significantly linked to both social isolation and fewer overall friendships. Regarding a broader concept of friendship, our results indicate that hyperactive or emotional symptoms by themselves did not affect the dynamics of a friendship in children who had at least one friend. However, variations in the conduct domain were noted [29]. In another study led by Wang and colleagues, more than a third (33.9%) of adolescents asserted experiencing the feeling of loneliness, which was correlated with greater total difficulty scores and decreased prosocial scores compared with their non-lonely peers [30].

Another crucial factor associated with the mental and emotional well-being of adolescents is bullying. Research indicates that bullying affects physical health and contributes to increases in the likelihood of experiencing mental health problems [31]. In 2019, the United Nations Educational, Scientific and Cultural Organization noted that the prevalence of bullying among adolescents was 32% [32]. According to a WHO report based on a survey conducted in 2021–2022, around one in ten children are bullied at school a minimum of two to three times per month. Major risk factors associated with higher bullying rates were socio-demographic characteristics such as living with other family members vs. both parents and parent’s education level, employment, and income. In general, approximately 20–25% of adolescents participate in bullying either as targets, perpetrators, or both. The school environment is one of the risk factors. Data suggest that the size of the class and classroom hierarchy can be associated with bullying prevalence. Bullying is more common in classes with fewer pupils and distinctly hierarchical classrooms, which means that higher status, such as popularity, belongs to a small group of peers and is not distributed evenly. Moreover, bullying is observed to be more frequent in classes where teachers do not stop bullying and other children do not defend victims or even uphold and join the bully [33]. Experiencing such violence is linked to a greater risk of mental health disorders, academic struggles, physical well-being problems, loneliness, anxiety, depression, and worse overall well-being. The psychological and physical damage caused by bullying leaves long-lasting consequences. The most vulnerable individuals are those who are both bullies and victims. They have the highest probability of experiencing psychiatric disorders later in life [34]. Mohseny’s study on Iranian students found substantial and positive associations between conduct, emotional, social, peer, and hyperactivity problems (SDQ items) and bullying. Notably, social challenges were significantly correlated only with bullying, not with victimization [35]. Therefore, it is vital to organize support to ensure that bullied children receive the help they need.

Research conducted by the World Health Organization in 2021–2022 revealed that Lithuania exhibits the highest incidence of bullying victimization. Among girls, the prevalence was from 3% at the age of 15 in Italy, Spain, and Portugal to 33% among Lithuanian 13-year-olds [36]. For boys, the percentages varied between 2% among 15-year-olds in Belgium (French-speaking region) and France and 34% of 11-year-olds in Lithuania [36]. The National Education Agency of Lithuania gathered adolescent data during the first half of 2019. The results showed that 54.7% of students gave a positive opinion about the school, 31.3% gave a negative opinion, and 14% had both positive and negative views. According to most students who provided negative opinions about the school, there was a poor emotional environment, a lack of activity variety, and lessons/activities were seen as irrelevant and uninteresting. Additionally, some students reported little attention and support given to each student; they did not like the school, did not feel safe at school, and disagreed with the school rules. This indicated that these students did not feel well at school [37]. Relationships with peers and teachers are particularly significant for young people with behavioral problems (internalizing or externalizing). Positive relationships are associated with improved academic and social/emotional outcomes, while conflicting relationships are linked to more negative outcomes [38]. However, our study observed that greater student feeling of teacher care had a more significant impact on externalized difficulties than internalized ones. This could be related to the fact that the role of teachers as assistants or supporters is integral for teenagers to encounter as little hyperactivity or conduct problems as possible. According to a study conducted in the United States of America, children who have conflictual relationships with their teachers exhibit higher levels of behavior problems in middle childhood compared to their counterparts with less conflictual teacher–child relationships [39].

Our study found no associations between students’ emotional health and the size of the city where they lived, despite the scientific literature finding diverse associations with regard to this relationship. A study conducted in northern Spain compared the mental well-being of adolescents in urban and rural settings, revealing that those in rural areas reported notably higher Health-Related Quality of Life (HRQoL) scores and longer durations of sleep at night. Additionally, urban adolescents achieved lower scores in the dimension related to the school environment (higher scores indicated more positive perception) [40]. Existing research suggests that adolescents residing in urban settings tend to face higher levels of academic anxiety, thereby influencing their engagement with the school environment [41]. Vytautas Magnus University in Lithuania has reported that individuals residing in large cities exhibit higher levels of happiness compared to residents of towns, small cities, and rural regions, including country villages, farms, and countryside homes. Additionally, city dwellers are more satisfied with their lives than residents of towns, minor urban regions, and countryside areas [42]. There are also known differences in the availability of psychological help between cities and countryside areas. Children residing in small and rural communities are at a higher risk of experiencing psycho-emotional, behavioral, and developmental disorders compared with those living in suburbs or cities. In rural areas, there exists a significant deficit of psychiatrists and psychologists with the capacity to ensure qualified assistance to youth who seek help [43].

## 5. Conclusions

Over the last decade, there has been a considerable surge in psychological health challenges affecting young people. Increasing numbers of specialists are encountering adolescents seeking help. It is essential to recognize the contributing factors and particular groups of teenagers who are in the most significant need of psycho-emotional support. According to our research, it has been established that girls are more predisposed than boys to encounter psychological and emotional difficulties. In addition, adolescents living with one parent experienced greater emotional and behavioral difficulties than those in two-parent families. This study found that only one in ten students sought external help for problems, feelings, behavior, or emotional troubles, and most tended to communicate with their peers. Loneliness, a feeling of safety at school, and bullying were associated with poorer psycho-emotional health in students. Students’ help-seeking was associated with gender and experiences of loneliness. The findings of this study may offer valuable insights for advancing additional prevention or support programs aimed at adolescents within higher-risk groups.

## 6. Limitations

When conducting an analysis of this study, it is imperative to take into account its multiple limitations. This study constitutes a cross-sectional analysis, facilitating the identification of associations between various factors. However, it does not provide a framework for testing causal relationships. It is conceivable that the presence of companionship and well-being factors within the school setting could influence the development of behavioral and emotional issues. Conversely, it is also plausible that adolescents facing specific emotional challenges may experience heightened feelings of insecurity or isolation within the school setting. We believe that longitudinal studies could more accurately evaluate the factors that most impact adolescents’ mental well-being. Another limitation is that our focus was solely on students aged 12–17 years in the study, which did not allow us to discern variations across childhood stages. Additionally, we utilized non-standardized tools to assess adolescents’ friendships and well-being at school, which is another aspect to consider.

## Figures and Tables

**Table 1:** Main sample characteristics.

Background Variable	N	%
**Gender**		
Boys	2037	50.9
Girls	1965	49.1
**The cities in which the students live**		
Large cities (population > 100,000)	1644	39.9
Cities that are district centers (population of 5000 to 100,000)	1318	32.0
Rural areas (population < 5000)	1162	28.1
**In which grade the adolescents study**		
7	952	23.9
8	986	24.8
9	1052	26.4
10	994	24.9
**Family composition**		
Family with both parents	3437	83.3
Family with one parent	592	14.4

**Table 2:** Characteristics of the students’ well-being at school and their friendships.

Variable	N	%
**Over the last 12 months, how frequently have you experienced feelings of loneliness?**		
Never	1080	26.6
Rarely	1145	28.2
Sometimes	998	24.5
Most of the time	644	15.8
Always	200	4.9
**How many close friends do you have?**		
Zero friends	178	4.4
Only one friend	424	10.4
Two friends	783	19.3
Three or more friends	2675	65.9
**How frequently in the last six months have you experienced bullying at school?**		
Not at all	3003	74.2
<1 per week	677	16.7
>1 per week	224	5.5
Most days	143	3.5
**I feel safe at school**		
Never	491	11.9
Sometimes	1058	25.7
Often	1382	33.5
Always	1193	28.9
**Teachers care about me**		
Never	1169	28.3
Sometimes	1504	36.5
Often	952	23.1
Always	499	12.1

**Table 3:** Emotional and behavioral problems among students compared across genders, cities of residence, and family compositions.

Background Variables	TDS		IS		ES	
	Mean	SD	Mean	SD	Mean	SD
**Gender**						
Male	12.45	5.39	5.39	3.30	7.07	3.14
Female	14.93	5.76	7.49	3.64	7.44	3.33
***p***	<0.001		<0.001		0.053	
The cities in which the students live *						
Large cities	13.91	3.72	6.53	3.59	7.38	3.23
Cities that are district centers	13.69	3.46	6.40	3.61	7.29	3.29
Rural areas	13.51	3.42	6.35	3.68	7.16	3.26
***p***	0.171		0.371		0.207	
Family with both parents	13.50	5.69	6.32	3.61	7.18	3.23
Family with one parent	14.76	5.72	6.98	3.65	7.78	3.35
***p***	<0.001		<0.001		<0.001	

TDS: total difficulty score (SDQ), IS: internalizing score (SDQ), ES: externalizing score (SDQ), SD: standard deviation. * In the comparison of the SDQ TDS, IS, and ES among the cities where the students studied, no statistically significant differences were discovered through the application of Tukey’s post hoc test.

**Table 4:** Comparison of students’ psycho-emotional problems and help-seeking behavior.

Within the Last Six Months, Have You Ever Felt the Need for Outside Help with Your Problems, Feelings, Behavior, or Emotional Troubles?									
	No, I Have Not Felt the Need		I Have Considered Seeking Outside Help		I Have Sought Outside Help				
	Mean	SD	Mean	SD	Mean	SD	*p*	Tukey’s Post Hoc Test	Correlation Coefficient
SDQ TDS	11.85	5.14	16.09	5.25	17.80	5.75	<0.001	1 < 2 < 3	0.405 **
SDQ IS	5.18	3.14	8.05	3.41	9.11	3.59	<0.001	1 < 2 < 3	0.430 **
SDQ ES	6.68	3.10	8.04	3.19	8.49	3.56	<0.001	1 < 2 < * 3	0.232 **

TDS: total difficulty score (SDQ), IS: internalizing score (SDQ), ES: externalizing score (SDQ), SD: standard deviation. * *p* > 0.05. ** *p* < 0.05.

**Table 5:** Comparison of students’ psycho-emotional problems and students’ well-being at school.

**How Frequently in the Last Six Months Have You Experienced Bullying at School?**											
	**Not at All**		**Less Than Once per Week**		**More Than Once per Week**		**Most Days**				
	**Mean**	**SD**	**Mean**	**SD**	**Mean**	**SD**	**Mean**	**SD**	***p***	**Tukey’s Post Hoc Test**	**Correlation Coefficient**
SDQ TDS	12.62	5.36	15.94	5.39	17.66	5.09	19.73	5.68	<0.001	1 < 2 < 3 < 4	0.328 **
SDQ IS	5.71	3.34	7.93	3.40	9.10	3.63	10.33	3.62	<0.001	1 < 2 < 3 < 4	0.341 **
SDQ ES	6.91	3.18	8.01	3.27	8.56	3.09	9.41	3.24	<0.001	1 < 2 < * 3 < * 4	0.197 **
**I Feel Safe at School**											
	**Never**		**Sometimes**		**Often**		**Always**				
	**Mean**	**SD**	**Mean**	**SD**	**Mean**	**SD**	**Mean**	**SD**	***p***	**Tukey’s Post Hoc Test**	**Correlation Coefficient**
SDQ TDS	17.61	6.17	15.94	5.23	13.00	4.90	11.01	5.21	<0.001	1 < 2 < 3 < 4	−0.397 **
SDQ IS	8.66	4.07	7.85	3.51	5.96	3.09	4.82	3.14	<0.001	1 < 2 < 3 < 4	−0.372 **
SDQ ES	8.95	3.37	8.09	3.16	7.04	3.00	6.19	3.14	<0.001	1 < 2 < 3 < 4	−0.285 **
**Teachers Care about Me**											
	**Never**		**Sometimes**		**Often**		**Always**				
	**Mean**	**SD**	**Mean**	**SD**	**Mean**	**SD**	**Mean**	**SD**	***p***	**Tukey’s Post Hoc Test**	**Correlation Coefficient**
SDQ TDS	16.05	5.90	14.05	5.178	11.98	5.07	10.63	5.61	<0.001	1 < 2 < 3 < 4	−0.321 **
SDQ IS	7.56	3.86	6.66	3.430	5.51	3.27	4.94	3.27	<0.001	1 < 2 < 3 < * 4	−0.247 **
SDQ ES	8.50	3.39	7.39	3.007	6.48	2.92	5.69	3.21	<0.001	1 < 2 < 3 < 4	−0.283 **

TDS: total difficulty score (SDQ), IS: internalizing score (SDQ), ES: externalizing score (SDQ), SD: standard deviation. * *p* > 0.05. ** *p* < 0.05.

**Table 6:** Comparison of students’ emotional and behavioral problems and students’ friendships.

**Over the Last 12 Months, How Frequently Have You Experienced Feelings of Loneliness?**													
	**Never**		**Rarely**		**Sometimes**		**Most of the Time**		**Always**				
	**Mean**	**SD**	**Mean**	**SD**	**Mean**	**SD**	**Mean**	**SD**	**Mean**	**SD**	***p***	**Tukey’s Post Hoc Test**	**Correlation Coefficient**
SDQ TDS	10.29	4.76	12.32	4.83	14.75	4.79	18.20	4.90	20.54	5.43	<0.001	1 < 2 < 3 < 4 < 5	0.520 **
SDQ IS	4.12	2.71	5.42	2.90	7.11	2.98	9.64	3.17	11.07	3.34	<0.001	1 < 2 < 3 < 4 < 5	0.568 **
SDQ ES	6.17	3.00	6.90	3.03	7.64	3.19	8.56	3.18	9.47	3.68	<0.001	1 < 2 < 3 < 4 < 5	0.285 **
**How Many Close Friends Do You Have?**													
	**0 Friends**		**1 Friend**		**2 Friends**		**3 or More Friends**						
	**Mean**	**SD**	**Mean**	**SD**	**Mean**	**SD**	**Mean**	**SD**			***p***	**Tukey’s Post Hoc Test**	**Correlation Coefficient**
SDQ TDS	17.76	6.05	15.88	5.93	14.69	5.71	12.82	5.43			<0.001	1 < 2 < 3 < 4	−0.224 **
SDQ IS	9.95	3.97	8.44	3.81	7.24	3.46	5.65	3.29			<0.001	1 < 2 < 3 < 4	−0.310 **
SDQ ES	7.81	3.41	7.43	3.43	7.44	3.35	7.17	3.20			0.053	1 < * 2 < * 3 < * 4	−0.047 **

TDS: total difficulty score (SDQ), IS: internalizing score (SDQ), ES: externalizing score (SDQ), SD: standard deviation. * *p* > 0.05. ** *p* < 0.05.

**Table 7:** Regression analysis of the SDQ TDS, SDQ IS, and SDQ ES.

	SDQ TDS			SDQ IS			SDQ ES		
	B	SE_b_	β	B	SE_b_	β	B	SE_b_	β
Intercept	10,879	0.563		4.308	0.339		6.571	0.379	
Gender	0.405	0.151	0.036 *	0.749	0.091	0.103 *	−0.344	0.102	−0.053 *
Cities	0.101	0.088	0.014	0.113	0.053	0.025	−0.012	0.059	−0.003
Bullying	1.290	0.102	0.166 *	0.839	0.061	0.170 *	0.451	0.069	0.102 *
A feeling of safety at school	−0.999	0.089	−0.173 *	−0.614	0.053	−0.167 *	−0.385	0.060	−0.117 *
A feeling of teacher care	−0.565	0.086	−0.097 *	−0.026	0.052	−0.007	−0.540	0.058	−0.163 *
Loneliness	1.528	0.074	0.314 *	1.069	0.045	0.345 *	0.459	0.050	0.166 *
Friends	−0.412	0.088	−0.061 *	−0.689	0.053	−0.160 *	−0.276	0.059	−0.072 *

TDS: total difficulty score (SDQ), IS: internalizing score (SDQ), ES: externalizing score (SDQ), B = unstandardized regression coefficient, SE_b_ = standardized error of the coefficient, and β = standardized coefficient. * *p* < 0.001.

**Table 8:** Factors associated with students’ help-seeking.

Variable	Odd Ratio	95% CI	*p*
Gender	2.30	1.97–2.69	<0.001
Cities	0.90	0.82–0.99	0.026
Bullying	1.09	0.98–1.22	0.108
A feeling of safety at school	1.05	0.95–1.15	0.322
A feeling of teacher care	1.08	0.98–1.19	0.108
Loneliness	1.77	1.63–1.91	<0.001
Friends	0.92	0.84–1.01	0.097
SDQ TDS	1.04	1.02–1.07	<0.001
SDQ IS	1.10	1.05–1.14	<0.001

TDS: total difficulty score (SDQ), IS: internalizing score (SDQ), CI: confidence interval.

## Data Availability

The raw data supporting the conclusions of this article will be made available by the authors without undue reservation.
